# Enhanced Mechanical and Corrosion Performance by Forming Micro Shear Bands in Cold Forged Mg-Gd-Y-Zr Alloy

**DOI:** 10.3390/ma13143181

**Published:** 2020-07-16

**Authors:** Zhengjiang Yang, Chuming Liu, Yonghao Gao, Xueyi Guo, Yingchun Wan

**Affiliations:** 1 School of Materials Science and Engineering, Central South University, Changsha 410083, China; zjyangcsu@126.com (Z.Y.); cmliu@csu.edu.cn (C.L.); 13874894140@126.com (Y.G.); 2 School of Metallurgy and Environment, Central South University, Changsha 410083, China; xueyiguo_csu@163.com

**Keywords:** magnesium alloy, micro shear bands, microstructure, mechanical properties, corrosion resistance

## Abstract

Forging at room temperature was applied on the per-extruded Mg-Gd-Y-Zr alloy to investigate the effect of cold forging on the microstructure, mechanical properties and corrosion resistance of the alloy. Abundant micro shear bands with misorientations of 2–15° were generated in the as forged alloys. Tremendous enhancement in tensile yield strength was achieved after forging. With a quantitative investigation, micro band boundaries were considered to provide a great contribution to the reinforcement. The ultrafine structure resulting from the formation of micro shear bands led to increased corrosion resistance of the alloy.

## 1. Introduction

Magnesium alloys are the most promising lightweight structural materials owing to their low density, high specific strength and ideal recyclability, etc. [1]. However, poor corrosion resistance limits their wide application in many fields. Many enhancement methods, such as the generation of precipitates usually result in sharp decrement in corrosion resistance by introducing particles of higher potential [2]. Recent investigations indicated that low temperature deformation was an alternative approach in enhancing Mg alloys. For example, after high pressure torsion, the ultrafine-grained Mg-Gd-Y-Nd-Zr alloy exhibited a yield strength of 450 MPa [3]. After rolling at room temperature, Mg-Gd-Y-Nd-Zn-Zr alloy exhibited a yield strength higher than 400 MPa with the generation of a large quantity of stacking faults (SFs) [4]. With the reinforcement introduced by the ultrafine structure, the as-forged AZ61 alloy yielded at a high strength of 480 MPa [5]. As the enhancement was not obtained through precipitation, it may be a good approach to develop an Mg alloy with both high mechanical properties and corrosion resistance. However, effect of cold deformation on corrosion resistance of Mg alloys is ambiguous and deserves systematic investigations. In addition, quantitative understanding of the underlying strengthening mechanism of cold deformed Mg alloy is of significant importance. For Mg and Mg alloys, cold deformations are mostly carried out via various kinds of forging, such as rotary swaging [6,7,8] and multi-directional forging [5,9]. Because the triaxial compressive stress condition of forging could help reduce crack and the strain rate can be better controlled, compared with rolling and extrusion.

Among the various recently developed Mg alloys, Mg-Gd-Y alloys exhibit comparatively good corrosion resistance and high strength, making them available in diverse applications [10,11,12]. However, in order to obtain a desired strength, Mg-Gd-Y-based alloys were usually subjected to ageing treatment to generate precipitates, which is proved have detrimental influences on both the ductility and the corrosion resistance. Developing a new approach to improve the strength with corrosion resistance maintained is essential for the application of Mg-Gd-Y alloys.

## 2. Experimental Procedure

The material used in the present study is the as-extruded Mg-8Gd-3Y-0.4Zr alloy. The forging was conducted at room temperature with a total area reduction of 12% after two passes (7% in the first pass). According to the strain level, the forged samples are named as F-7 for 7% strain and F-12 for 12% strain, respectively. The as used material is named AE.

The microstructure was examined by a Leica optical microscope (OM) (Buffalo Grove, IL, USA), HELIOS Nano Lab 600i Dual Beam Electron Microscope for EBSD observation (FEI, Hillsboro, OR, USA), and FEI Tecnai G2 F-120 and Titan G2 60-300 transmission electron microscope (TEM) operated at 200 Kv. The X-ray micro strain analysis of the alloy was analyzed using a Bruker D8 X-ray diffractometer (Billerica, MA, USA). The test power was 3 kW, using Cu-Kα radiation source. The scanning speed was 0.02°/min with a scanning range of 10–80°.

Tension tests were conducted using an Instron 3369 tester following the ASTM standard B557M-94 under the strain rate of 10^−3^/s at room temperature. Specimens for tension were ultimately machined to a dog-bone shape possessing the gauge diameter of 5 mm and gauge length of 25 mm. Three parallel samples were used for the results.

All of the electrochemical measurements were carried out in 0.35% NaCl solution at room temperature using an IM6 Zahner-electrik Gmbh electrochemical workstation (Kansas City, MO, USA). The samples used for electrochemical test were prepared with an exposed area of 8 × 8 mm^2^ with the surfaces grinded using 2000-meshed SiC abrasive paper. A standard three-electrode setup was used to perform electrochemical measurement: a test sample, a platinum wire and a saturated calomel electrode (SCE) acting as working electrode, counter electrode and reference electrode. The open circuit potential (OCP) measurement was set for 600 s. The potentiodynamic polarization (PDP) curves were carried out from −0.2 to 0.4 V (vs. SCE) at a scanning rate of 0.5 mV/s. Four replicates for each condition were used to ensure reproducibility.

## 3. Results 

### 3.1. Microstructure Evolution 

**Figure 1:**
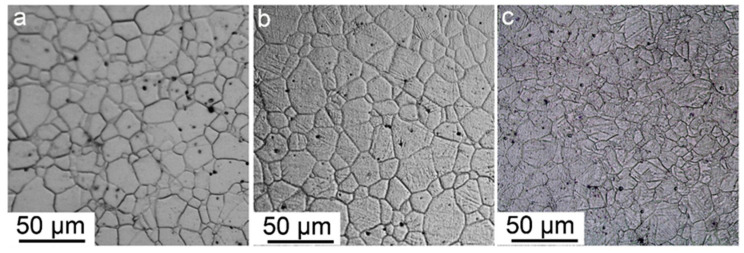
Optical microscope structure of different samples (**a**) AE; (**b**) F-7; (**c**) F-12.

Figure 1 shows the OM microstructure of the alloys in the as extruded and forged conditions. The as extruded alloy, as shown in Figure 1a, was characterized with nearly equiaxial grains with average size of 10 μm. It is clean both in the grain interior and at grain boundary, without obvious second phase particles witnessed. This morphology indicates full recrystallization without occurrence of dynamic precipitation. After the alloy was forged to a strain level of 7%, as shown in Figure 1b, the grains did not exhibit obvious change in size or shape compared with sample AE, except for the appearance of a small quantity of twin. With the strain level elevated, the grain boundaries lost their clarity and sharpness after etched in the same solution and for the same duration. However, in regions of identical areas, their grain numbers are nearly the same, verifying approximate grain sizes among the three samples. 

**Figure 2:**
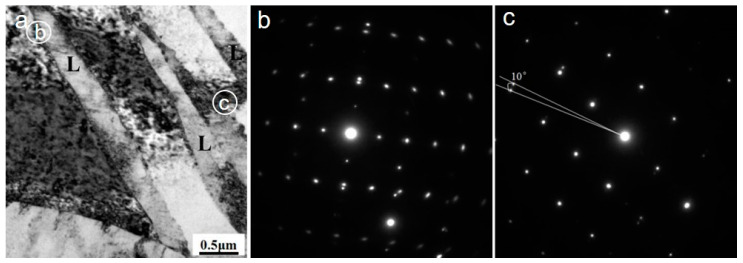
Transmission electron microscopy (TEM) images of sample F-12. (**a**) bright field image; (**b**) selected diffraction pattern of region “b” in (**a**); (**c**) selected diffraction pattern of region “c” in (**a**).

Figure 2 shows the TEM microstructure and selected area electron diffraction (SAED) pattern of the alloy with a total strain of 7%. Lamellas (indicated as “L”) with dimension of about 100–600 nm in thickness, 1–3 μm in length, and 0.5–1.5 μm in adjacent spacing were obtained as well as some randomly distributed dislocation cells. The SAED patterns of the lamellas reveal that both deformation twin and micro shear bands were obtained. The micro shear bands have misorientations varying from 2° to 10° with the matrix. Dislocations were observed at the both sides of the micro bands, revealing their formation is strongly related to dislocations gliding. The micro shear bands observed here are different from the macro shear bands usually observed in hot processed Mg alloy extending across several grains or even nearly the whole processed samples. They are considered to be formed through the sever deformation on the shear plane along the shear direction of sample via twining or twining dynamic recrystallization [13,14,15,16]. The heavy traces along basal plane and curved feature of the dislocations indicate that the dislocations glide on both the basal and non-basal planes.

**Figure 3:**
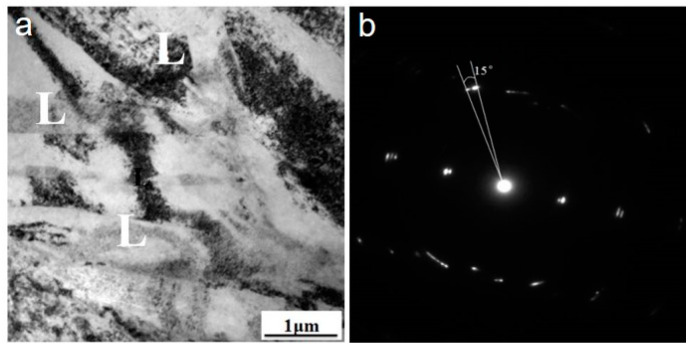
Transmission electron microscopy images of sample F-12. (**a**) bright field imagee; (**b**) selected oodiffraction pattern of a micro shear band.

Figure 3 shows the TEM microstructure and SAED diffraction pattern of the alloy with a total strain of 12%. Compared with sample F-7, more densely distributed lamellas were obtained. The SAED pattern reveals that micro shear bands with misorientation of 15° began to appear, indicating the misorientation of the micro shear bands could be increased with strain. The increment in misorientation was thought a result of dislocation multiplication at the pre-formed band boundaries. In addition, intersection of band-band, band-twin, and twin-twin were observed.

**Figure 4:**
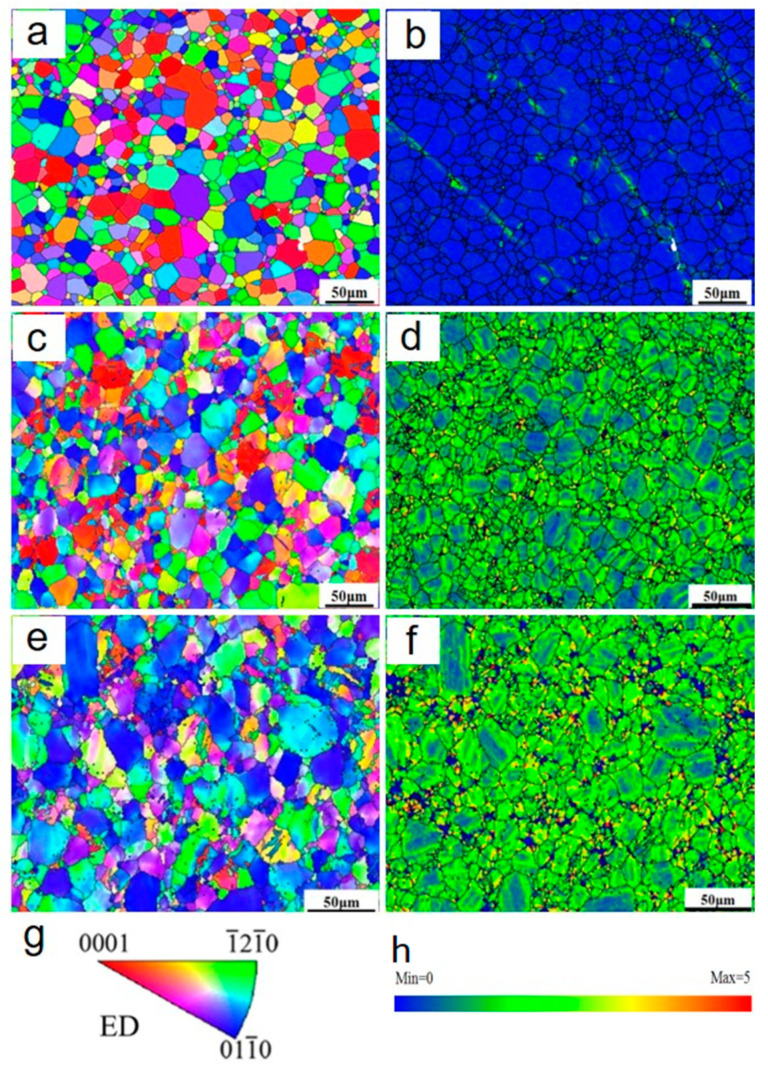
Inverse pole figure maps and local misorientation maps of various samples: (**a**,**b**) AE; (**c**,**d**) F-7; (**e**,**f**) F-12; (**g**) sample orientation; (**h**) local misorientation scale bar.

**Figure 5:**
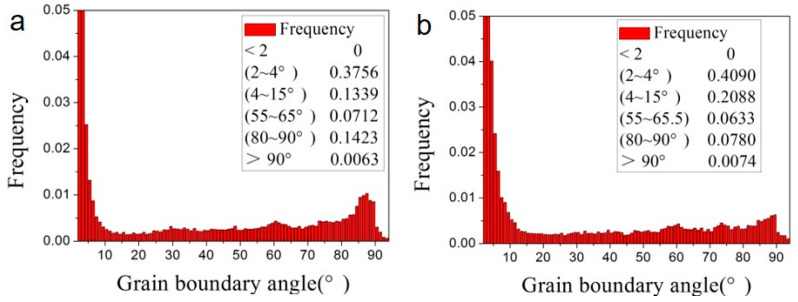
Misorientation distributions of various samples: (**a**) F-7; (**b**) F-12.

**Figure 6:**
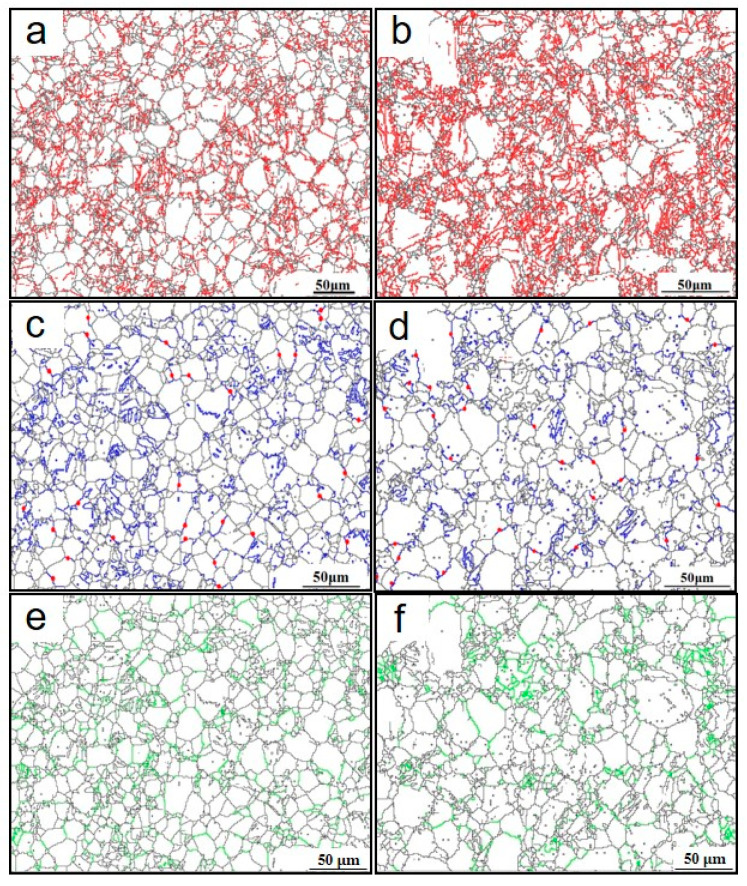
Misorientation distributions of various samples: (**a**,**c**,**e**) F-7; (**b**,**d**,**f**) F-12.

To further investigate the microstructure evolution, EBSD characterization was carried out and the results are shown in Figure 4, Figure 5 and Figure 6 and Table 1. The inverse pole figure in Figure 4a,c,e reveal that in the whole the grains are mostly re-oriented with the c axis parallel with the loading direction after forging, compared with the nearly random distribution in the as-extruded alloy. This feature of micro texture was obtained by twining at the beginning of deformation [17,18]. Local misorientation (LM) maps, usually used to evaluate the dislocation density of the deformed alloys, are shown in Figure 4b,d,f. The increased LM from grain interior to grain boundary indicates an increment in dislocation density.

The misorientation distributions of the forged alloy are shown in Figure 5 and Figure 6. For both the two as forged samples, the misorientation profile has three peaks, respectively at 2°–15°, 55°–65°, and 80°–90°, which are considered to correspond to the micro shear bands, {10$\bar{1}$1} twining and {10$\bar{1}$2} twining. The micro shear bands (indicated by the red line in Figure 6a,b were mostly observed near the grain boundaries, which were considered a result of stronger dislocation activity and inhomogeneous gliding near the grain boundaries. {10$\bar{1}$2} twining, indicated by the blue line in Figure 6c,d, has a strong dependence on the original grain orientation—that is, most of the {10$\bar{1}$2} twin was observed in the grains with {0001} plane parallel with the loading direction, while the {10$\bar{1}$1} twining (indicated by the green line in Figure 6e,f reveals an alignment with the micro shear bands or LM in addition to the grain orientation. In addition, the frequency ratio of {10$\bar{1}$1} twinning to {10$\bar{1}$2} twining increased with strain: 0.50 (0.0712/0.1423 = 0.50) for sample F-7 and 0.81 (0.0633/0.0780 = 0.81) for sample F-12. This feature indicates {10$\bar{1}$1} twinning is more frequently activated in grains with higher strain. It is generally considered that twin nucleates, grows and propagates by twining dislocation activity. The value of twin dislocation burgers vector is higher for {10$\bar{1}$1} twin than {10$\bar{1}$2} twin [19,20], and higher energy is required for the nucleation and growth of {10$\bar{1}$1} twin. Only when the grains are pre-hardened by dislocations inhomogeneous gliding, strain high enough for {10$\bar{1}$1} twinning will be achieved. As LM in wrought metals represents dislocation and micro strain level, {10$\bar{1}$1} twin has an alignment with LM.

### 3.2. Mechanical Properties and Strengthening Mechanism

Effect of cold forging on mechanical properties of the alloy was studied. Table 2 gives the strength and elongation to failure of the samples. Increments of yield strength are 66 MPa and 135 MPa, respectively for sample F-7 and F-12. 

Texture is usually considered to have great influence on the mechanical behavior of Mg alloy when the deformation is accommodated in grain interior, due to the high differences in the critical resolved shear stress for twining systems and various dislocation slipping systems. In the present study, the samples are all characterized with weak basal texture as shown in the inverse pole figures in Figure 4. Thus, influence of texture on strength of the alloy can be ignored here. Twin boundaries have high mobility under strain in grains at the micrometer scale and are considered to have very minor strengthening effects. Thus, the strengthening is considered to result from the deformation-generated dislocations.

**Figure 7:**
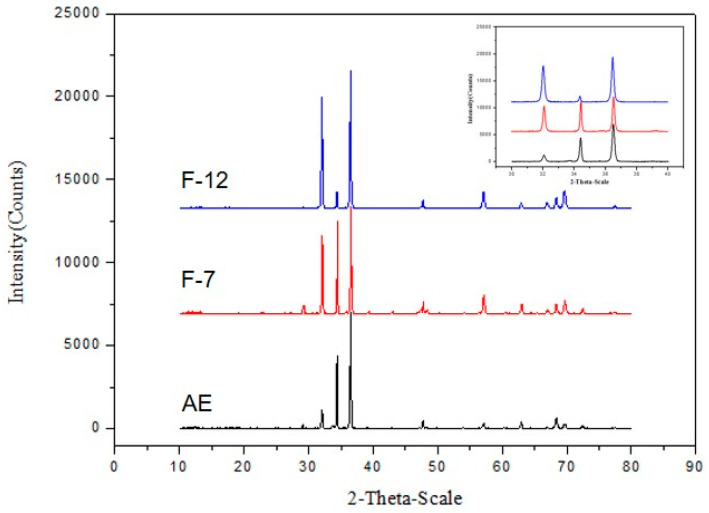
X-ray diffraction pattern of different samples.

In the cold forged alloy, strengthening effect of dislocations can be evaluated based on the Bailey–Hirsch Equation (1),

Δσ = MaGbρ^1/2^ (1)

where A is 0.24 [14,15,16], M is Taylor factor, 2.1 for magnesium dislocations basal slip, G is the shear modulus of the material, and ρ is the dislocation density. X-ray diffraction (shown in Figure 7) was carried out to measure the micro strain <ε^2^>^1/2^ and evaluate the dislocation density based on the broadening of the peaks for {0002}, {10$\bar{1}$0}, {11$\bar{2}$0} planes. Equation (2) was used here [17,18]:

(2) $$\rho =\frac{2\surd 3{<\varepsilon^{2}>}^{1/2}}{D\times b}$$

where *b* is the Burgers vector, which is 3.21 × 10^−10^ m. *D* is grain size. The calculated dislocation density and strengthening contribution are summarized in Table 3.

Comparing the calculated values with the experimental ones, we can conclude that the obtained strengthening effects are higher than the calculated ones. The additional strengthening is considered to be caused by the micro shear bands. 

The micro shear bands are thought to be generated via parallel dislocations by forming low angle boundaries in Ta and Nb [21,22]. The parallel dislocations have different contribution to the mechanical behaviors of the alloy, compared with the randomly distributed ones. When dislocations glide inside a grain which is free of micro shear bands, some of them are tracked, intersected and interacted with others, while others could still glide without being hindered. While when dislocations glide within grains with high density of bands, nearly all of them will be tracked and interacted with the band boundaries. Then material will be much quickly hardened, exhibiting higher stress. When the stress is high enough for the dislocations to pass the band boundaries, the material will be softened, and the stress will go down immediately. Therefore, the alloy with micro shear bands exhibited higher strength but weakened hardening ability.

**Figure 8:**
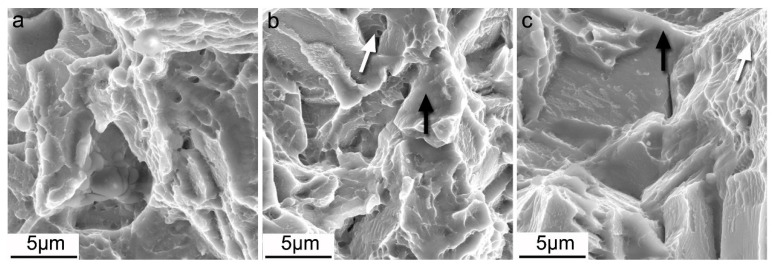
Fracture surfaces different samples: (**a**) AE; (**b**) F-7; (**c**) F-12.

Figure 8 shows the SEM fracture surface morphology of the as extruded and as forged samples. On the fracture surface of sample E, as shown in Figure 8a, dimples and cleavages were witnessed, revealing a mixed fracture mechanism operated. Along with the strain level, the fracture surface morphology began to exhibit differences. As shown in Figure 8b, the fracture surface of sample F-7 was composed of deep dimples as indicated by the white solid arrow, and a large quantity of cleavage plane as indicated by the black solid arrow. The cleavage planes were of lamellar shape, which were believed to correspond to the twins and micro shear bands. Figure 8c shows the morphology of sample F-12 with a fracture surface composed of tearing bands as indicated by the white solid arrow, and smooth cleavage planes, as indicated by the black solid arrow. The morphologies of the as extruded and as forged alloys indicate that for all of the samples the crack propagate interior of the grains. A cleavage fracture takes increasing fraction, and the ductility decreases with elevated strain level, coincidently matching well with the tension results.

## 4. Corrosion Resistance

**Figure 9:**
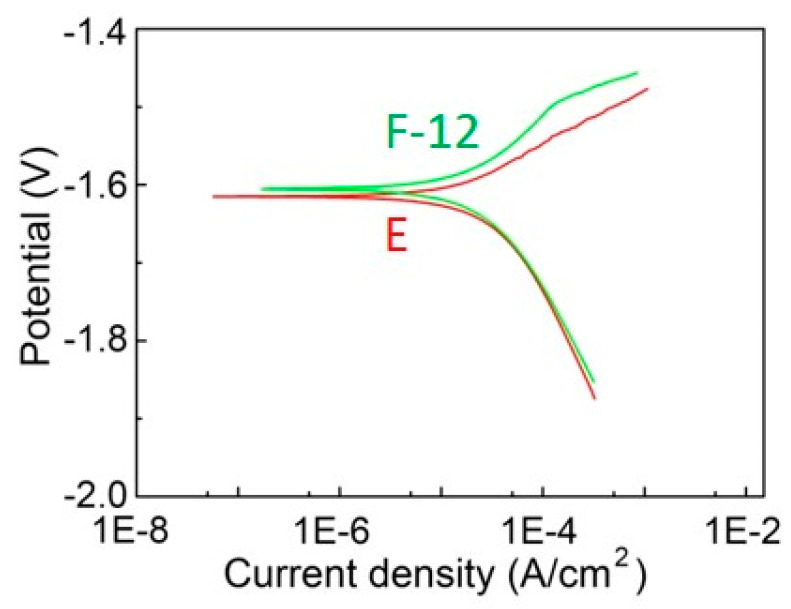
Polarization curves of the alloy.

Figure 9 gives the corrosion properties of the alloy before and after forging. The polarization curves revealed similar cathodic kinetics but slightly reduced anodic kinetics, implying a reduced corrosion rate was obtained in the forged alloy. The slightly positively moved corrosion potential (E_corr_) and reduced corrosion current intensity (I_corr_), as summarized in Table 4, confirmed the enhanced corrosion resistance.

The notable enhancement in corrosion resistance is considered as a result of the banded structure obtained through forging. The extremely refined grains are usually considered to benefit the decrease of the corrosion rate by improving the stability of the protective oxide (MgO) film formed on the substrate. When the oxide film forms, the free volume mismatch will arise and introduced cracks or ruptures [20]. It is believed that the increased density of grain boundary could provide a route to relieve the stress through enhanced atomic flow along the grain boundaries in the substrate. Thereby, the oxide layer could adhere better to the substrate and help to enhance the alloy’s corrosion resistance. 

## 5. Conclusions

In this study, we investigated the evolution of microstructure, mechanical properties and corrosion resistance of Mg-Gd-Y-Zr (wt.%) alloy during cold forging by conducting TEM, SEM and EBSD characterizations, tension tests and polarization measurements. A large quantity of micro shear bands was generated after cold forging. The micro shear bands are of hundreds of nanometers in thickness and with misorientations of 2°–15° with the matrix. Tremendous enhancement of the strength was obtained due to the formation of micro shear bands. Structure refinement induced by micro shear bands also help improving the alloy’s corrosion resistance. 

## Figures and Tables

**Table 1:** The distributions of grain boundary angle of various samples.

Samples	Misorientation		
	(2°–15°)	(55°–65°)	(80°–90°)
F-7	0.50	0.0712	0.1423
F-12	0.61	0.0633	0.0780

**Table 2:** Mechanical properties of the alloy.

Samples	Yield Strength (MPa)	Ultimate Tensile Strength (MPa)	δ (%)
AE	200 (6.1)	310 (5.3)	19 (1)
F-7	266 (2)	367 (4.4)	9 (2)
F-12	335 (4.4)	397 (2.6)	5 (1)

**Table 3:** Contribution of work hardening to the yield strength of the alloy.

Samples	Grain Size/μm	Micro Strain/%	Dislocation Density/m^−2^	Work Hardening/MPa
F-7	12	0.163	0.047 × 10^16^	58
F-12	12	0.249	0.072 × 10^16^	72

**Table 4:** Corrosion properties of the alloy.

Sample	I_corr_ (10^−5^ A/cm^2^)	E_corr_ (V)
E	2.630	−1.6178
F-12	2.294	−1.6071
